# Does coevolution with a shared parasite drive hosts to partition their defences among species?

**DOI:** 10.1098/rspb.2017.0272

**Published:** 2017-05-17

**Authors:** Eleanor M. Caves, Martin Stevens, Claire N. Spottiswoode

**Affiliations:** 1 Department of Zoology, University of Cambridge, Downing Street, Cambridge CB2 3EJ, UK; 2 Centre for Ecology and Conservation, College of Life and Environmental Sciences, University of Exeter, Penryn Campus, Penryn, Cornwall TR10 9FE, UK; 3 DST-NRF Centre of Excellence at the FitzPatrick Institute, University of Cape Town, Rondebosch 7701, South Africa

**Keywords:** brood parasitism, colour, mimicry, coevolution, phenotypic space

## Abstract

When mimicry imposes costs on models, selection may drive the model's phenotype to evolve away from its mimic. For example, brood parasitism often drives hosts to diversify in egg appearance among females within a species, making mimetic parasitic eggs easier to detect. However, when a single parasite species exploits multiple host species, parasitism could also drive host egg evolution away from other co-occurring hosts, to escape susceptibility to their respective mimics. This hypothesis predicts that sympatric hosts of the same parasite should partition egg phenotypic space (defined by egg colour, luminance and pattern) among species to avoid one another. We show that eggs of warbler species parasitized by the cuckoo finch *Anomalospiza imberbis* in Zambia partition phenotypic space much more distinctly than do eggs of sympatric but unparasitized warblers. Correspondingly, cuckoo finch host-races better match their own specialist host than other local host species. In the weaver family, parasitized by the diederik cuckoo *Chrysococcyx caprius*, by contrast, parasitized species were more closely related and overlapped extensively in phenotypic space; correspondingly, cuckoos did not match their own host better than others. These results suggest that coevolutionary arms races between hosts and parasites may be shaped by the wider community context in which they unfold.

## 1. Introduction

When mimicry is costly to models, selection should drive a model's phenotype to evolve away from that of its mimic [1]. Such models include the vertebrate immune system [2,3], and the hosts of reproductive parasites including insects [4] and birds [5]. Hosts of avian brood parasites lay their eggs in the nests of other birds, and rely on deception such as egg and chick mimicry to fool the host parents into providing costly care to their young [6]. In several independent brood-parasitic systems, hosts have defended themselves by diversifying their own egg phenotypes away from those of parasites, resulting in egg ‘signatures’ that help hosts to detect mimetic parasitic eggs [7–9]. However, many brood-parasitic species have evolved multiple sympatric host-races that specialize on different hosts and show appropriate egg mimicry for each [10–12]. If individuals of a host escaping parasitism diversify their egg signatures into phenotypic space occupied by another host, they may become susceptible to pre-existing mimicry by another parasitic host-race if attempts at host-switching occur [11]. Consequently, we should expect hosts not only to be more diverse in appearance than unparasitized species [7,13–15], but specifically to diversify egg phenotypes away from those of other sympatric hosts.

In a two-host system with sympatric hosts H_a_ and H_b_, parasitized by parasites P_a_ and P_b_, respectively, host H_a_ can escape from mimicry by parasite P_a_ by shifting its own egg phenotype away from P_a_. But if it shifts its phenotype into the area of phenotypic space occupied by host H_b_, it risks parasitism from parasite P_b_, so long as P_b_ is capable of switching to a new host. Adding other sympatric hosts and parasites further restricts areas of unoccupied phenotypic space. Therefore, selection should favour host individuals that reduce phenotypic overlap with hosts of other parasites, because they will be susceptible to a smaller subset of the parasitic population. There is evidence from numerous brood-parasitic systems that host-switching by parasites is a relevant selection pressure on hosts. Specialist parasites sometimes lay eggs in the nest of the wrong species; for example 12.1% of eggs laid by common cuckoos (*n* = 1397; [16]) were non-mimetic for the host species in whose nest they were laid, and 1.8% of nests parasitized by cuckoo finches *A. imberbis* (*n* = 276; C.N.S. 2017, unpublished data) belonged to a different species from that mimicked by the parasitic egg; both figures are probably underestimates as many mismatched eggs will have been rejected by the hosts before the nest was found. Correspondingly, host-switches have repeatedly occurred over evolutionary time, leading to the evolution of new parasitic species [17] or host-races [18,19]; indeed many host colonization events must have once begun with such events.

Parasites, in turn, are under selection to track their respective hosts through phenotypic space. As a result, greater phenotypic partitioning among hosts should drive greater specialization among parasitic host-races, resulting in parasites better matching the eggs of their own specific host than those of other co-occurring hosts. However, if hosts do not partition phenotypic space then parasites may correspondingly overlap with many hosts and operate more like generalists.

This coevolutionary scenario makes clear predictions both for the degree of phenotypic partitioning among groups of sympatric hosts, and between host–parasite pairs. The hypothesis that hosts experience selection from multiple specialized host-races of parasites, favouring phenotypic partitioning among hosts, predicts (i) that host egg phenotypes of different species should be more distinct from one another than are the egg phenotypes of related, sympatric species that are not exploited by brood parasites. If so, then (ii) parasitic egg phenotypes should be more similar to those of the host species they were found in (hereafter ‘own host’) than to those of other hosts. Alternatively, if host species overlap extensively with one another, then parasites need not match their own host any more closely than they match other hosts, and parasitized and unparasitized species should show similar levels of phenotypic partitioning.

Here, we test these predictions in two African brood-parasitic systems with different distributions of parasitism among hosts. The African warblers (Cisticolidae) parasitized by cuckoo finches provide a strong test of both predictions because parasitized and unparasitized species are dispersed across their phylogeny [14]. The weaverbirds (Ploceidae) parasitized by the diederik cuckoo *Chrysococcyx caprius* provide an interesting comparison: host species have variable but (to the human eye) largely overlapping egg phenotypes. However, only four species in our weaver dataset are unparasitized, two of which are in relatively distantly related, basal genera [20], preventing a strong comparison with parasitized species. Nevertheless, this system provides a good test of the second prediction, that close host–parasite matching is only expected when hosts are phenotypically distinct.

First, for each family we quantified the degree of phenotypic partitioning within each group of sympatric host species and compared it to that found in co-occurring species that are not currently parasitized in the study area. Second, we examined the consequent degree of phenotypic specialization of parasites both to their own host and to other hosts. We measured phenotypic partitioning and mimicry in multi-dimensional space, because egg signatures are comprised of multiple traits such as colour, luminance (perceived lightness) and pattern, which hosts are known to integrate when making rejection decisions [11,21]. The hypothesis assumes that parasites occasionally lay an egg in the nest of a species other than their usual specialist host, which is known for at least the cuckoo finch system (above); it also assumes that host egg appearance is not solely explained by phylogenetic relatedness, which we test.

## 2. Material and methods

### (a) Study system

**Figure 1.**
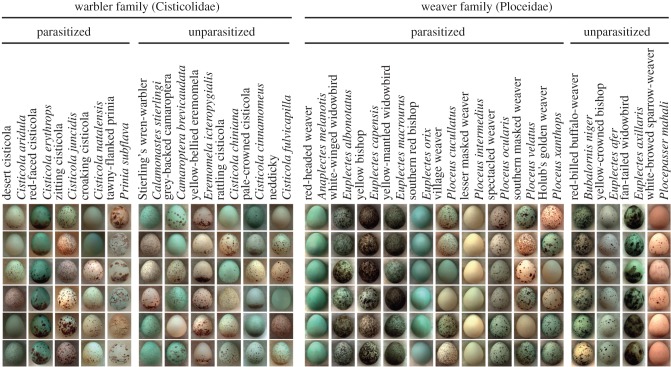
Representative egg phenotypes for each of the parasitized and unparasitized warbler (Cisticolidae) and weaver (Ploceidae) species in this study. Each egg is from a different clutch. (Online version in colour.)

Within each bird family, some host species show marked inter-clutch variation in appearance (egg ‘signatures’; figure 1), which is at least partially matched by corresponding variation within parasitic host-races [11,22]. In each system, different parasitic host-races, their respective hosts and unparasitized species of the same family all occur sympatrically and in similar habitats in the study area [11,22]. We treated each family separately, because they make slightly different predictions (see §1) and because parasites are highly unlikely to switch between host families owing to differences in body size, habitat and timing of breeding. We measured eggs in the private collection of Major John Colebrook-Robjent (bequeathed to the Natural History Museum, Tring, United Kingdom), which were all collected in the Choma, Monze and Mazabuka Districts (primarily Choma, 16°47′ S, 26°50′ E) of southern Zambia from 1970–1990. Our dataset comprised 939 clutches from 11 warbler species (five parasitized, six unparasitized), 14 weaver species (10 parasitized, four unparasitized), five parasitic host-races of the cuckoo finch, and five parasitic host-races of the diederik cuckoo (details in [14]). We randomly selected one egg per clutch for analysis to avoid pseudoreplication.

### (b) Quantifying egg phenotypes

We used reflectance spectra to quantify egg colour and digital photography to quantify egg pattern, following the methods reported in [14]. Briefly, we calculated photon catches for the double cones, and the UV, SW, MW and LW single cones, which we used as indices of luminance and colour, respectively [9]. We applied a granularity approach [23] to digital photographs to quantify five pattern traits, as previously used to quantify egg pattern [9,11,24]: predominant marking size, contribution of the main marking to overall pattern, contrast between pattern markings and background, the proportion of the egg's surface covered by markings, and dispersion of markings across the egg.

### (c) Discriminant function analysis

To quantify and visualize partitioning in phenotypic space, we used discriminant function analysis (DFA [25]; for an excellent description see [26]). First, DFA generates discriminant functions, which are linear combinations of classification variables that maximize the probability of correctly assigning observations to their pre-determined groups. Second, DFA can classify each observation into one of the groups, and assess the success rate of classification. Mathematically, DFA is identical to a single-factor MANOVA; however, whereas MANOVA tests hypotheses about what factors underlie group differences, DFA emphasizes classification and prediction of group membership [26]. Here, we used DFA to yield an index of the degree of partitioning between species in phenotypic space by quantifying the degree to which species (group membership) are discriminable by linear combinations of the colour and pattern traits defined above (our classification variables) [27,28]. The discriminant rate represents the percentage of observations (i.e. eggs) assigned to the correct species based on a set of classification variables (i.e. egg phenotypic attributes). Higher accuracy of DFA (i.e. higher percentage of eggs classified to the correct species or host-race) reflects less phenotypic overlap, and thus greater phenotypic partitioning, among groups.

DFA is sensitive to variation in sample size [26,29,30]; it may be more likely to correctly assign an egg to the correct species by chance if it is represented by many clutches, or if the analysis comprises few species. Additionally, in the classification step DFA classifies the same observations used to generate the classification functions [26]. We therefore used jack-knifing to estimate the accuracy of the discriminant rate [31,32]: one observation in the sample is omitted, a discriminant function calculated, and the omitted observation is categorized. This is repeated with each observation omitted in turn [33]. As a separate confirmation of the discriminant rate, we repeated our analyses where possible using a Monte Carlo sample splitting approach [34], which in all cases yielded results that were of the same direction and significance as jack-knifing (methods and results in electronic supplementary material, table S1).

DFA can be sensitive to non-normality, collinearity and heterogeneity of variances [33,35]. We examined normality using normal q–q plots, and used Mahalanobis distances to identify and remove outliers from the dataset [29,36,37]; we found and removed one outlier from our warbler dataset (a *Cisticola chiniana* egg), and five outliers from our weaver dataset (all *Euplectes orix* eggs). We used Pearson correlation coefficients to identify collinear pairs of variables. In both warblers and weavers, the photon catch pairs UV-MW and SW-LW were highly correlated (*r* > 0.8; see [26], Chapter 5, pp. 72–110); therefore, we repeated all analyses with one variable from each pair (LW and MW) removed. We used the arcsine-square-root transformation to reduce heterogeneity of variances among test groups, and repeated all analyses on the transformed data. Removing correlated variables and transforming the data did not change the direction or significance of our conclusions in any instance (electronic supplementary material, tables S2 and S3); therefore the results in the main text are from untransformed data with correlated traits not removed.

To further guard against any violations of DFA's fairly restrictive assumptions, we also performed a multinomial logistic regression, which can be used to characterize observations when the response variable has more than two categories. In general, logistic regression makes fewer assumptions than DFA, but is less powerful when sample sizes are small, and when all of the assumptions of DFA are met [38]. Results are given alongside those of the DFA for our ‘groupwise’ analyses (see below).

#### (i) Using discriminant function analysis to examine phenotypic partitioning among sympatric hosts and non-hosts

We carried out DFA in two ways for each bird family. First, we conducted ‘groupwise’ analyses, in which we compared the accuracy (i.e. discriminant rate) of phenotypic partitioning among parasitized species, to that among unparasitized species. This gives an overall measure of phenotypic partitioning within a group of species. We used jack-knifing (above) to help to control for differences in sample size of clutches between species within a group. However, there were also different numbers of species within each group of parasitized and unparasitized species. Therefore, we also conducted a ‘pairwise’ analysis in which we compared the accuracy (i.e. discriminant rate) of phenotypic partitioning among all possible pairs of parasitized species, to that among all possible pairs of unparasitized species. These analyses provide a less realistic picture of how a community of species responds to parasitism pressure, but have the advantage of removing any bias introduced by differences between groups made up of different sample sizes of species.

Before analysis, we calculated a null hypothesis of classification rates based solely on the relative sample sizes of species within each group; therefore, this ‘expected accuracy’ was the probability that a species would be correctly assigned due to chance alone. First, this tested the assumption that DFA performs better than chance in classifying species. Second, it allowed us to apply each of our groupwise and pairwise analyses to the ‘expected’ data as well as to the ‘observed’ data. To reject our null hypothesis, an effect of parasitism status should be present in the observed classification rates (i.e. based on phenotypic traits) that is not also present in the expected classification rates (i.e. based on chance alone arising from sample size variation).

As the groupwise analysis yielded a point estimate of observed and expected classification rates, respectively, we compared them using Fisher's exact tests and calculated their binominal proportion confidence intervals [39]. The pairwise analysis yielded a distribution of observed and expected classification rates, which we compared using Welch's (unequal variances) *t*-tests on ranked data [40]. We did not use Bonferroni or other similar methods to correct for multiple testing, as each test generated a discriminant rate not a *p*-value, and therefore was not a significance test.

#### (ii) Using discriminant function analysis to examine host–parasite similarity

To test whether a parasitic host-race is phenotypically more similar to its own host than to other hosts, we performed DFA between a given parasitic host-race and its own host, and between it and each other host species in turn. For each host-race, DFA yielded a measure of accuracy for an ‘own’ comparison, as well as multiple accuracies for ‘other’ comparisons. Within a given host-race, we subtracted the ‘other’ value from the ‘own’ value and took the mean of those differences to yield an average measure of how much more phenotypically similar a host-race is to its own host than other hosts. To assess significance, we used a paired *t*-test to examine the differences between ‘own’ and ‘other’ comparisons within each host-race. For the cuckoo finch, sample size for two of five host-races was very low (*Cisticola erythrops, n* = 2; *C. natalensis*, *n* = 1). Therefore, these eggs were not included in statistical analyses, but are presented in the figures for completeness.

### (d) Phylogenetic methods

To test the extent to which phylogenetic relationships [41] may have confounded the DFA, we estimated the degree of phylogenetic signal exhibited by each trait individually within each family. We used Pagel's *λ* [42] to estimate the extent to which variation in a given trait is explained by phylogenetic structure, ranging from zero (no phylogenetic signal) to one (variation completely explained by phylogenetic structure). Some species in our study have either not been formally placed on a tree, or placed but with low confidence; to address this uncertainty, we used birdtree.org [43] to compile 100 trees with branch lengths for each focal family.

We then used the R package caper [44] to calculate phylogenetic signal (Pagel's *λ*) in each of the 100 trees per family. For each tree, we calculated Pagel's *λ*, as well as *p*-values for significance tests of whether *λ* differed significantly from zero (*p*_0_, which would indicate significant phylogenetic signal) or one (*p*_1_, which would indicate no significant phylogenetic signal). We then calculated the average and standard deviation of *λ*, *p*_0_ and *p*_1_ for each trait. We found no evidence of significant phylogenetic signal in egg traits in the Cisticolidae (electronic supplementary material, table S4), as in all cases lambda differed significantly from one, but not from zero. However, in the Ploceidae, we found that both luminance (*p*_0_ = 1.00 ± 0.00, *p*_1_ = 0.10 ± 0.28) and UV (*p*_0_ = 1.00 ± 0.00, *p*_1_ = 0.08 ± 0.23) did not differ significantly from either zero or one. This indicates that phylogenetic structure is neither helpful nor unhelpful in explaining the trait distribution; this likely occurred because the phylogeny is small and, due to phylogenetic uncertainty, has large confidence intervals. However, low values for Pagel's *λ* (*λ* = 0.15 ± 0.32 for luminance and *λ* = 0.08 ± 0.26 for UV) suggest that the influence of phylogeny on luminance and UV is not of large magnitude. Complete results of the phylogenetic signal analyses are in electronic supplementary material, table S4.

## 3. Results

### (a) Does discriminant function analysis separate species better than chance?

Within each group (parasitized and unparasitized warblers and weavers), observed accuracy of DFA based on phenotypic traits was significantly higher than expected accuracy based on chance alone. This indicates that irrespective of parasitism status, DFA performed significantly better than chance at classifying individuals to species (comparisons within columns in table 1). Similarly, in the pairwise analyses, observed accuracy within a group was always significantly higher than expected accuracy (*t*-test, *p* < 0.0001 in all cases). This, first, justified the use of DFA to quantify phenotypic partitioning between species within groups and, second, generated expected classification rates that could be applied to each analysis below, for comparison with observed classification rates.

**Table 1.** Accuracy, expected correct, observed correct and improvement over chance in categorzing eggs to species using both jack-knife validation of discriminant function analysis, and multinomial logistic regression, within groups of parasitized and unparasitized warblers and weavers.

	warbler family		weaver family	
	parasitized species (*n* = 205 clutches, 5 species)	unparasitized species (*n* = 219 clutches, 6 species)	parasitized species (*n* = 339 clutches, 10 species)	unparasitized species (*n* = 46 clutches, 4 species)
discriminant function analysis (DFA)				
accuracy ± 95% CI (%)	82.4 ± 5.21	54.8 ± 6.59	63.7 ± 5.12	100 ± 0.00
expected correct	44	42	46	12
observed correct	169	120	216	46
*p* (expected versus observed)^a^	<0.001	<0.001	<0.001	<0.001
improvement over chance	60.9%	36.6%	50.2%	74.6%
multinomial logistic regression				
accuracy (%)	92.2	66.2	79.4	100
expected correct	44	42	46	12
observed correct	189	145	269	46
*p* (expected versus observed)^a^	<0.001	<0.001	<0.001	<0.001
improvement over chance	70.7%	47.0%	65.8%	73.9%

^a^Fisher's exact test.

### (b) Phenotypic partitioning in warblers (Cisticolidae)

**Figure 2.**
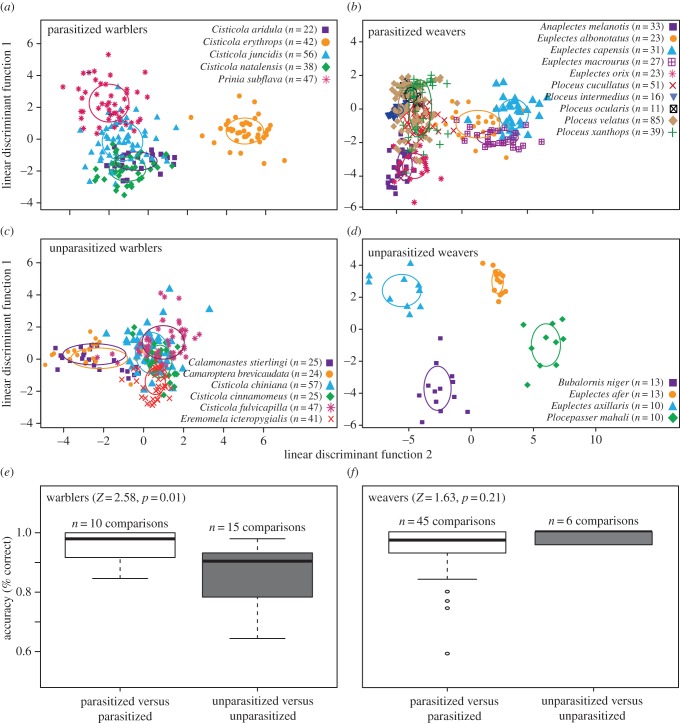
Phenotypic partitioning between parasitized and unparasitized warbler and weaver species. Plots (*a–d*) show the first two linear discriminant function scores for (*a*) parasitized and (*c*) unparasitized warbler species, and (*b*) parasitized and (*d*) unparasitized weavers. The height and width of the ellipses around each group centroid represent one standard deviation of discriminant function 1 and discriminant function 2, respectively; the contributions of different phenotypic variables to the two linear discriminant functions are given in electronic supplementary material, table S5. Plots (*e*) and (*f*) show the accuracy (the percentage of observations categorized correctly) of pairwise DFAs between either pairs of parasitized species or pairs of unparasitized species. Higher accuracy indicates that species are, on average, more phenotypically distinct from one another and thus have less phenotypic partitioning. Statistics are from unequal variances (Welch's) *t*-tests on ranked data. Taken together, the results show that parasitized warblers partition phenotypic space more strongly among species than do unparasitized warblers, but that there is no clear difference in phenotypic partitioning between parasitized and unparasitized weaver species. (Online version in colour.)

Parasitized and unparasitized groups of species did not differ in expected accuracy, i.e. the likelihood of correctly classifying eggs based purely on chance given differences in sample size between groups (Fisher's exact test, *p* = 0.55; comparisons across columns in table 1). However, once we incorporated phenotypic information, using either a DFA or logistic regression, observed accuracy was significantly higher for parasitized than unparasitized warblers (Fisher's exact test, *p* < 0.0001 for both DFA and logistic regression; comparisons across columns in table 1). Taken together, this indicates that DFA is indeed better able to distinguish among parasitized species than among unparasitized species (figure 2*a,b*), and that this is not simply an artefact of different sample sizes within and between groups, or of the statistical approach used. Correspondingly, in the pairwise comparisons, based on chance there was no significant difference in expected accuracy between pairs of parasitized (mean ± s.e. = 52.9 ± 0.01 per cent) and unparasitized species (53.3 ± 0.01 per cent; *Z* = 1.00, *p* = 0.32). Incorporating phenotypic traits, pairs of parasitized species were significantly more accurately classified (95.6 ± 1.7 per cent accurate) than pairs of unparasitized species (86.1 ± 2.6 per cent; *Z* = 2.58, *p* = 0.01). Thus, DFA was better able to distinguish pairs of parasitized species than pairs of unparasitized species (figure 2*a,b*). In summary, the results consistently supported our prediction of greater than expected phenotypic partitioning among parasitized warblers than unparasitized warblers.

### (c) Phenotypic partitioning in weavers (Ploceidae)

The small number of unparasitized weaver species (*n* = 4) in the dataset undermines comparisons with parasitized species, and correspondingly we found that higher accuracy was expected for unparasitized species than for parasitized species, based purely on chance (Fisher's exact test, *p* = 0.045; comparisons across columns in table 1). Similarly, when phenotypic information was incorporated, via either DFA or logistic regression, observed accuracy was significantly higher for unparasitized than parasitized species (figure 2*b,d*; Fisher's exact test, *p* < 0.001 for both DFA and logistic regression). Thus, the null hypothesis was not rejected, because we found the same result in the expected accuracy based on chance alone as well as based on the observed phenotypic data, probably because both suffered from the same bias, making it difficult to draw clear conclusions. In the pairwise comparisons (figure 2*f*), the expected pattern went in the opposite direction: expected accuracy was significantly higher for pairs of parasitized species (59.1 ± 0.03 per cent) than pairs of unparasitized species (52.2 ± 0.03 per cent; *Z* = −4.26, *p* < 0.001). This discrepancy with the groupwise analysis is explained by a different kind of bias: although it is no longer relevant that sample size differs between groups, expected accuracy differs within groups because sample sizes are more variable among parasitized weaver species (yielding > 50% accuracy by chance) than among unparasitized weaver species (yielding approximately 50% accuracy by chance). When we incorporated phenotypic information, there was no significant difference in the accuracy of classification between pairs of parasitized species (95.1 ± 1.5 per cent) than between pairs of unparasitized species (98.8 ± 0.02 per cent; *Z* = 1.63, *p* = 0.21; figure 2*f*) This indicates that pairs of parasitized species were no more or less discriminable from one another than were pairs of unparasitized species, despite being better discriminated based on their phenotypes than expected by chance. In summary, in the weavers we were unable to robustly reject our null hypothesis, as the groupwise analysis of the observed data yielded the same pattern as expected by chance, and because the pairwise results did not detect a significant difference in discriminability between parasitized and unparasitized species.

### (d) Do parasites match their own host better than other hosts?

**Figure 3.**
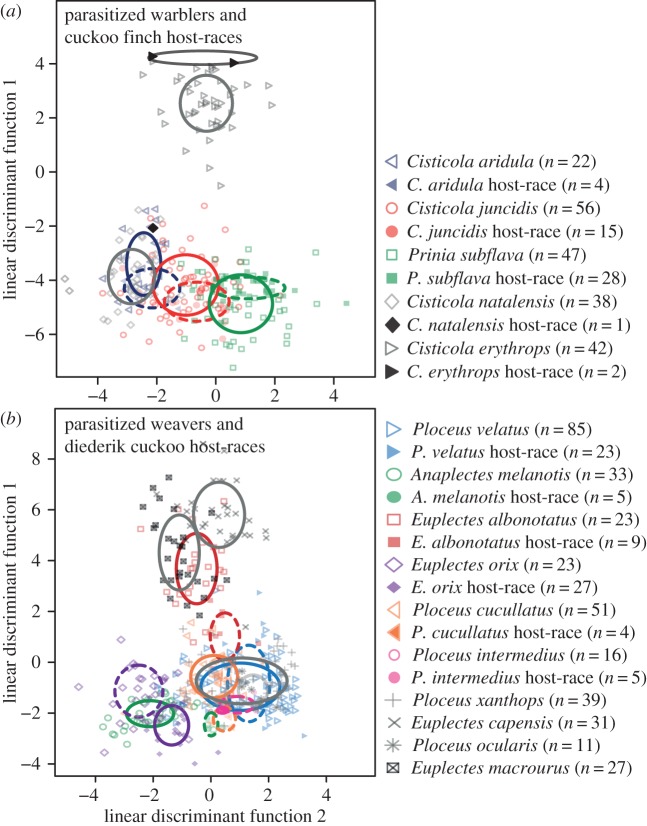
Phenotypic specialization of (*a*) cuckoo finch host-races to warbler host species and (*b*) diederik cuckoo host-races to weaver host species. In the warblers, parasitic host-races are significantly better visual mimics of their own host species than of other co-occurring host species; by contrast, in the weavers, parasitic host-races are no better mimics of their own host species than of other hosts (see the §3d for statistical analyses). In both panels, a host species and its parasitic host-race are represented by the same shape and colour, host species with hollow symbols and parasitic host-races with filled symbols. Host species in grey indicate species for which we had little or no data regarding parasitic host-races. In panel (*a*), we had very low sample sizes of parasitic eggs for the parasitic host-races that parasitize *C. erythrops* (*n* = 2) and *C. natalensis* (*n* = 1); thus, we did not include these data points in statistical analysis, but show them here (in black) for completeness. The height and width of the ellipses around each group centroid represent one standard deviation of discriminant function 1 and discriminant function 2, respectively; ellipses around host-races are solid, while those around parasitic host-races are dashed. (Online version in colour.)

The above results predict that host–parasite matching should occur in the cuckoo finch–warbler system, but not in the diederik cuckoo–weaver system. Both predictions were supported: in the warbler family, we found that DFA was significantly less accurate at discriminating between a cuckoo finch host-race and its corresponding host species, than between the same host-race and other host species (figure 3*a*). Comparisons between cuckoo finch host-races and their own host were on average 10.5 ± 2.43 per cent less accurate than comparisons between cuckoo finch host-races and other host species (*t*_2_ = 4.31, *p* = 0.02). In the weaver family, comparisons between diederik cuckoo host-races and their own host (figure 3*b*) were only 3.9 ± 1.34 per cent less accurate than comparisons between diederik cuckoo host-races and other weaver hosts, and this difference was not significant (*t*_4_ = 3.32, *p* = 0.06).

## 4. Discussion

Distinguishing self from non-self is paramount to the hosts of avian brood parasites, as it is to the victims of many other aggressive mimics. To improve their chances of detecting a parasitic mimic, hosts can diversify their own eggs into a multi-dimensional phenotypic space comprised of such traits as egg colour, luminance and pattern [8,9,14]. In this study, we asked whether such diversification by different host species can be constrained by susceptibility to other parasitic strains, such that co-occurring host species might indirectly shape one another's coevolutionary trajectories with a shared parasitic species.

In support of this hypothesis, we found that sympatric warbler host species of the cuckoo finch are phenotypically less similar to each other than are sympatric, unparasitized warbler species (figure 2*e*). Thus, hosts partition egg phenotypic space much more distinctly than do related species that are not currently parasitized. Because of the high level of phylogenetic relatedness among the parasitized warblers (four of the five unparasitized species are in the genus *Cisticola*), they would in the absence of parasitism likely be more phenotypically similar to each other simply as a result of shared phylogenetic history. The fact that they are statistically more discriminable than unparasitized warblers despite their relatedness lends strength to this result, especially given that the unparasitized warblers come from four different genera. As predicted for groups of hosts that partition phenotypic space among themselves, we found that cuckoo finch host-races more closely matched their own warbler host species than other co-occurring warbler hosts, supporting a second prediction of this coevolutionary scenario.

By contrast, in the weaver family, which are hosts of the diederik cuckoo, many host species have diverse eggs, and this diversity overlaps among species. Correspondingly, we were unable to reject our null hypothesis of no difference with unparasitized species. As predicted given this lack of phenotypic partitioning, diederik cuckoo host-races were on average not specialist mimics of their own host, such that parasitic eggs found in the nests of a given host species sometimes spanned the phenotypic space of several other host species. Others have previously noted this in South Africa and speculated that a single host-race may exploit multiple *Ploceus* species that each lays highly diverse eggs [45].

This invites the question of why weaver hosts, unlike warbler hosts, do not partition phenotypic space among closely related species, contrary to our prediction that selection should drive them to diversify between as well as among species. We have already underlined that the four species of unparasitized weaver are a poor ‘control’ group, owing to their small number and greater phylogenetic distance. However, there are also important ecological differences between systems that may explain why parasitized weavers tended to overlap in egg phenotype variation. First, weavers may experience weaker selection from host-switches than do warblers, if switches are rarer because hosts differ more in ecological traits such as nest architecture [46] and timing of breeding [22], or if their respective parasites differ in the traits used to recognize or locate their hosts. Second, weavers may experience weaker selection from brood parasites at the egg stage, if their front-line defences against laying cuckoos are superior owing to communal vigilance and nest defence in colonial species [47]. Third, intra-specific brood parasitism is common among weavers, which may select for phenotypic diversity within a species irrespective of interspecific parasitism, and thus confound any signal of cuckoo parasitism [48]. Finally, we might speculate that the arms race between weavers and the diederik cuckoo may be younger than that occurring between warblers and the cuckoo finch, which is known to be an exceptionally ancient species [49]; greater coevolutionary advancement should be associated with more sophisticated host defence. Each of these potential explanations might add noise to our results, blurring differences among groups of hosts and non-hosts.

Much research on species interactions has focused on cases where the relationship is mediated by a single trait in each species (reviewed in [50]). In nature, however, the majority of antagonistic interactions between species are governed by multiple traits [51–53]. For example, wild parsnip resistance to parasitic webworms is influenced by both flowering phenology and at least two different chemical defence compounds [54], and parasitism by monogenean and copepod parasites on teleost fish is mediated by both mucosal barriers and biocidal secretions [55]. In the brood-parasitic systems in this study, host defence is based on multiple visual traits that parasites need to mimic adequately in order to be accepted [11,21], and we therefore tested our predictions in a multi-dimensional trait space comprised of colour, luminance and pattern. This is important because theoretical work has shown that hosts can achieve an advantage over their parasites when host–parasite coevolution is mediated by multiple traits, and that the host's advantage increases as the number of traits governing the system increases [52]. This arises because successful parasites must overcome all of the defences produced by a host, lending hosts more options for escaping from parasitism. Such theoretical work underlines that parasites may find it easiest to switch between hosts with similar defensive phenotypes, and especially when such host defences comprise multiple traits and hence are hardest to overcome. Work with gallwasps (Cynipidae) lends support to this hypothesis, as specialized parasitoid wasps are more likely to switch between gallwasp hosts that induce phenotypically similar galls [56].

In summary, our results suggest that the evolution of signature-like defences against parasitism may be tempered by susceptibility to closely related parasitic strains. Similar processes might shape other antagonistic interactions where distinguishing self from non-self is crucial and has led to signature-like diversification in host traits, such as olfactory signatures in the hosts of insect social parasites [4], and molecular signatures in the adaptive immune system [2]. In support of recent calls to consider the community context of coevolutionary interactions [57,58], our results imply that when multiple host and parasitic lineages coexist, host–host interactions must be considered in tandem with host–parasite interactions to obtain a complete picture of the selection pressures driving host defences.

## Supplementary Material

Electronic Supplementary Material

## Data accessibility

Data are available from the Dryad Digital Repository: http://dx.doi.org/10.5061/dryad.dg58v [59].
