# Supplementary material for: Does coevolution with a shared parasite drive hosts to partition their defences among species?
Source: Proc Biol Sci. 2017 May 17;284(1854):20170272. doi: 10.1098/rspb.2017.0272 (PMC5443948; doi:10.1098/rspb.2017.0272)
Supplement: Electronic Supplementary Material [file rspb20170272supp1.docx]

Supplementary Materials for

**Does coevolution with parasites drive multiple hosts to partition their defences among species?**

Eleanor M. Caves, Martin Stevens, and Claire N. Spottiswoode

*Proceedings of the Royal Society B*

Article DOI: 10.1098/rspb.2017.0272

**Table S1. Monte-Carlo Split-Sample Cross Validation.** Because there is increasing evidence that different methods of validation for DFA can yield different results, we repeated our DFA between groups of parasitised and unparasitised warblers and weavers using a monte-carlo split sample cross validation to estimate the accuracy of our DFA, and to confirm the results found using the jackknife technique (results presented in Main Text). In monte-carlo split-sample cross validation the data are randomly split 50/50 into two subsamples, a training set and a validation set. The training set is then used to compute the discriminant function, and the accuracy of the discriminant function is assessed by classifying the individuals in the validation set (as in [1]). The procedure is repeated for 1000 permutations, and an overall correct classification rate is calculated. This table details the results of Monte-Carlo split sample cross-validation for a dataset that included untransformed data and did not exclude correlated variables, which were in every case very similar to the results from the jackknife approach.

|  | Parasitised Warblers  (n=205) | Unparasitised Warblers  (n=219) | Parasitised Weavers (n=339) | Unparasitised Weavers  (n=46) |
| --- | --- | --- | --- | --- |
| *Accuracy ± 95% C.I. (%)* | 78.2 ± 5.65 | 51.8 ± 6.62 | 57.6 ± 5.26 | 93.2 ± 7.28 |
| *Expected Correct* | 44 | 42 | 46 | 12 |
| *Observed Correct* | 160 | 114 | 203 | 43 |
| *Improvement over chance* | 56.5% | 35.3% | 46.5% | 67.2% |

**Table S2.** Degree of phenotypic specialization of parasites to hosts, using the jackknife method, and repeating analyses with LW and MW cone catches removed (to account for high correlatedness between variables) and with data transformed using Sqrt(Arcsin) to reduce heterogeneity of variances. The direction of the results did not differ significantly in either case presented here, from those presented in the main text.

|  | LW and MW Cone Catches Removed | | Sqrt(Arcsin) Transformation | |
| --- | --- | --- | --- | --- |
|  | Warblers | Weavers | Warblers | Weavers |
| Difference in Percent Accuracy Between “own” and “other” | 12.9 ± 4.72 | 4.28 ± 2.41 | 10.3 ± 3.89 | 2.89 ± 1.66 |
| Paired T-test (Average Other versus Average Own) | t_2_=2.748, p=0.05 | t_5_=1.612, p=0.12 | t_2_=2.69, p=0.5 | t_5_=1.235, p=0.09 |

**Table S3**. Jackknife and split-sample Monte Carlo Discriminant Function Analysis in parasitised and unparasitised warblers and weavers, first with LW and MW cone catches removed (to account for high correlation between variables), and second with data transformed with the Sqrt(Arcsin) transformation to reduce heterogeneity of variances. The direction and significance of these additional analyses are the same as the results presented in the main text. For both cone catches removed, and data transformed, Fisher’s exact tests showed that DFA correctly categorized individuals to species significantly more often than chance would predict for both parasitised and unparasitised species (p<0.001). In the warblers, there was no significant difference between parasitised and unparasitised species in the expected accuracy of DFA, but observed accuracy in parasitised species was significantly higher than that in unparasitised species (Fisher’s Exact Test, p<0.001 in all cases). In the warblers, however, the difference between parasitised and unparasitised weavers in the expected accuracy of DFA was marginally significant (Fisher’s Exact Test, p=0.045). Observed accuracy of DFA was significantly higher in parasitised weavers than in unparasitised weavers (Fisher’s Exact Test, p<0.001).

|  | LW and MW Cone Catches Removed | | | | Sqrt(Arcsin) Transformation | | | | |
| --- | --- | --- | --- | --- | --- | --- | --- | --- | --- |
|  | Parasitised Warblers  (n=205) | Unparasitised Warblers  (n=219) | Parasitised Weavers (n=339) | Unparasitised Weavers  (n=46) | Parasitised Warblers  (n=205) | Unparasitised Warblers  (n=219) | Parasitised Weavers (n=339) | Unparasitised Weavers  (n=46) |  |
| Jackknife |  |  |  |  |  |  |  |  |  |
| *Accuracy ± 95% C.I. (%)* | 78.5 ± 5.62 | 54.7 ± 6.59 | 56.6 ± 4.21 | 97.8 ± 4.21 | 82.4 ± 5.21 | 52.1 ± 5.21 | 63.7 ± 5.15 | 95.6 ± 5.90 |  |
| *Expected Correct* | 44 | 42 | 46 | 12 | 44 | 42 | 46 | 12 |  |
| *Observed Correct* | 161 | 120 | 191 | 44 | 169 | 114 | 215 | 43 |  |
| *Improvement over chance* | 57.0% | 36.1% | 43.1% | 71.8% | 60.9% | 33.4% | 50.2% | 69.6% |  |
|  |  |  |  |  |  |  |  |  |  |
| Monte-Carlo Split Sample |  |  |  |  |  |  |  |  |  |
| *Accuracy ± 95% C.I. (%)* | 77.7 ± 5.70 | 48.7 ± 6.62 | 54.1 ± 6.18 | 94.3 ± 6.21 | 78.4 ± 5.63 | 54.6 ± 6.59 | 62.2 ± 5.24 | 92.7 ± 7.58 |  |
| *Expected Correct* | 44 | 42 | 46 | 12 | 44 | 42 | 46 | 12 |  |
| *Observed Correct* | 159 | 107 | 183 | 43 | 161 | 120 | 210 | 42 |  |
| *Improvement over chance* | 56.2% | 30.0% | 40.6% | 68.6% | 56.9% | 35.9% | 48.7% | 66.7% |  |

Table S4: Tests for phylogenetic signal, in traits used in Discriminant Function Analysis. Pagel’s *λ* was calculated using the R package caper, as were *p(λ_0_)* and *p(λ_1_)*, which are significance tests of whether *λ* differs from 0 and 1, respectively. Because *λ, p(λ_0_),* and *p(λ_0_)* were calculated for each of 100 trees for each family, the values shown below are mean ± standard deviation.

|  | Proportion Energy | Total Power | Marking Filter Size | Proportion Coverage | Dispersion | Luminance | UV | SW | MW | LW |
| --- | --- | --- | --- | --- | --- | --- | --- | --- | --- | --- |
| Warblers |  |  |  |  |  |  |  |  |  |  |
| *λ* | 0.00±0.00 | 0.00±0.00 | 0.00±0.00 | 0.02±0.14 | 0.00±0.00 | 0.00±0.00 | 0.00±0.00 | 0.00±0.00 | 0.00±0.00 | 0.00±0.00 |
| *p(λ_0_)* | 1.00±0.00 | 1.00±0.00 | 1.00±0.00 | 1.00±0.00 | 1.00±0.00 | 1.00±0.00 | 1.00±0.00 | 1.00±0.00 | 1.00±0.00 | 1.00±0.00 |
| *p(λ_1_)* | 0.02±0.03 | 0.02±0.04 | 0.03±0.04 | 0.01±0.23 | 0.03±0.01 | 0.03±0.12 | 0.05±0.18 | 0.01±0.15 | 0.05±0.13 | 0.05±0.36 |
| Weavers |  |  |  |  |  |  |  |  |  |  |
| *λ* | 0.00±0.00 | 0.00±0.00 | 0.00±0.00 | 0.00±0.00 | 0.00±0.00 | 0.15±0.32 | 0.08±0.26 | 0.00±0.00 | 0.06±0.22 | 0.00±0.00 |
| *p(λ_0_)* | 1.00±0.00 | 1.00±0.00 | 1.00±0.00 | 1.00±0.00 | 1.00±0.00 | 1.00±0.00 | 1.00±0.00 | 1.00±0.00 | 1.00±0.00 | 1.00±0.00 |
| *p(λ_1_)* | 0.01±0.01 | 0.01±0.01 | 0.01±0.01 | 0.05±0.05 | 0.04±0.36 | 0.10±0.28 | 0.08±0.23 | 0.04±0.06 | 0.02±0.22 | 0.01±0.01 |

**Table S5:** Proportion of trace and standardized coefficients for Linear Discriminant Function 1/Linear Discriminant Function 2 in parasitized and unparasitized warblers and weavers. Proportion of trace represents the proportion of between-group variance that is explained by the linear discriminant function. The standardized coefficient for each variable represents the contribution of each variable to Linear Discriminant Function 1, i.e. how important that variable is in discriminating between groups. The greater the magnitude of the coefficient, the greater that variable’s contribution to discriminating between groups. In a discriminant function, the direction of the sign for each coefficient is arbitrary; thus, reversing the signs would result in no change in the interpretation of the results [2].

|  | Parasitised Warblers | Unparasitised Warblers | Parasitised Weavers | Unparasitised Weavers |
| --- | --- | --- | --- | --- |
| Proportion of Trace | 69.2/21.9 | 60.33/23.8 | 75.2/13.2 | 63.2/21.2 |
| Proportion Energy  *Relative contribution of main marking to overall pattern* | -0.04/-5.04 | 0.53/-0.35 | -1.49/3.56 | 4.49/-6.02 |
| Total Power  *A measure of contrast between egg pattern markings and background colour* | -0.76/3.50 | -2.62/3.61 | -2.61/-0.08 | -5.52/-3.84 |
| Marking Filter Size  *The predominant marking size* | -1.01/-3.74 | -2.70/-0.73 | 2.34/1.85 | 1.16/-0.72 |
| Proportion Coverage  *Proportion of egg covered with markings* | -2.03/2.46 | -0.92/4.20 | 5.96/2.06 | -11.9/-2.03 |
| Dispersion  *A measure of marking dispersal across the egg* | 2.41/1.28 | 0.82/-6.05 | -0.76/0.27 | 1.90/-5.23 |
| Luminance | -12.8/3.62 | 0.81/2.38 | -7.43/4.62 | -8.32/8.30 |
| UV cone catch | 18.2/-23.5 | 31.0/17.2 | 65.8/4.59 | 1.02/-30.4 |
| Short-wave cone catch | 13.6/-16.1 | -90.7/-84.8 | -121/-8.45 | 8.66/41.7 |
| Medium-wave cone catch | 27.8/33.0 | 82.1/123 | 202/-27.2 | -105/20.3 |
| Long-wave cone catch | -26.6/21.0 | -28.2/-30.4 | -85.6/15.0 | 41.3/23.6 |

**Literature Cited in the Supplementary Material**

1. Dechaume-Moncharmont, F., Monceau, K. & Cezilly, F. 2011 Sexing birds using discriminant function analysis: a critical appraisal. *Auk* **128**, 78–86.

2. Quinn, G. P. & Keough, M. J. 2002 *Experimental design and data analysis for biologists*. 1st edn. Cambridge University Press.
